# Regulatory Role of B Cells and Its Subsets in Hepatitis E Virus Infection

**DOI:** 10.1155/2022/7932150

**Published:** 2022-09-12

**Authors:** Meenal Sharma, Anuradha S. Tripathy

**Affiliations:** Hepatitis Group, ICMR-National Institute of Virology, Pune, 130/1, Sus Road, Pashan, 411021, Pune, Maharashtra, India

## Abstract

Antibodies as well as memory B cells are the potential correlates of a protective immune response against hepatitis E virus (HEV) infection. Literature on the role of B regulatory cells (Bregs) in acute viral infections is limited. We have evaluated the role of IL-10 expressing Bregs in HEV infection. A total of 108 acute hepatitis E patients, 55 hepatitis E recovered individuals and 128 HEV naïve healthy controls were enrolled. The percentages of peripheral CD19^+^, immature CD19^+^CD24^hi^CD38^hi^, mature CD19^+^CD24^int^CD38^int^ and memory CD19^+^CD24^hi^CD38^−^ B cells were analyzed by flowcytometry. Intracellular cytokine staining for IL-10 and TGF-*β*, HEV-rORF2p specific T cell response (IFN-*γ* expression) pre/post IL-10/IL-10R blocking and CD19^+^IL-10^+^ B cells-depletion based assays were carried out to assess the functionality of Bregs. The percentage of HEV-rORF2p specific immature B cell phenotype was significantly higher in acute hepatitis E patients compared to hepatitis E recovered individuals and controls. Significantly higher IL-10 expression on B and HEV-rORF2p stimulated immature B cells of acute hepatitis E patients compared to controls indicated that Bregs are functional and HEV-rORF2p specific. Enhanced IFN-*γ* expression on CD8^+^ T cells upon IL-10/IL-10R blocking and also post CD19^+^IL-10^+^ B cells depletion suggested that CD3^+^CD8^+^IFN-*γ*^+^ T cells corroborate the regulatory potential of Bregs via IL-10 dependent mechanism. We have identified HEV specific functional, immature CD19^+^CD24^hi^CD38^hi^ B cells having IL-10 mediated regulatory activities and a potential to modulate IFN-*γ* mediated T cell response in Hepatitis E. The prognostic/pathogenic role of Bregs in recovery from severe hepatitis E needs evaluation.

## 1. Introduction

Hepatitis E, a liver disease caused by hepatitis E virus (HEV) has emerged as a global health challenge, contributing to over 50% of the acute viral hepatitis cases in the endemic areas [1]. Though majority of patients with HEV infection follow a self-limiting course, very few develop severe form of hepatitis that may progress to fulminant hepatic failure (FHF) with a mortality rate of 20-30% in the third trimester of pregnancy. Chronic HEV infection and associated complications are also reported in immunocompromised and organ transplant recipients [2]. A remarkable deficit in knowledge still remains with respect to hepatitis E disease pathogenesis and control.

Association of B cells with antibody production and humoral immunity is a classic phenomenon. Robust antibody response to HEV-open reading frame 2 (HEV-ORF2) protein in the recovered individuals from HEV infection has been the basis for the development of vaccine against hepatitis E [3]. B cells are typically characterized as antibody producing cells and are known for their ability to function as secondary antigen presenting cells. However, one of their subsets defined as ‘B regulatory cells' (Bregs), having regulatory potential have emerged in the recent years [4]. The population of Bregs is relatively small (~less than 10%) under physiological conditions, but show substantial expansion in both patients and murine models of chronic inflammatory diseases, autoimmune diseases, infection, transplantation and cancer [5–8]. Bregs studies in human immunodeficiency virus (HIV) [9] and hepatitis B virus (HBV) infections [4] have indicated that the regulatory activities of Bregs are majorly interleukin-10 (IL-10) cytokine mediated, however immune regulation by production of transforming growth factor-beta (TGF-*β*) [10] and interleukin-35 (IL-35) [11] cytokines have also been reported. Phenotypically, subsets of Bregs have been defined as CD19^+^CD24^hi^CD38^hi^ immature/transitional B cells, CD19^+^CD24^int^CD38^int^ as mature B cells and CD19^+^CD24^int^CD38^−^ as memory B cells [12–14].

Our previous studies have reported higher levels of regulatory T cells (Tregs) and enhanced IL-10 and TGF-*β* cytokine production in acute HEV infection [15]. Subsequently, Tregs were found to be functional and exhibited TGF-*β* mediated suppressive activity [16]. Bregs are actively involved in inhibition of Th1 cells activation, Th17 cells differentiation and promotion, and maintenance of the Tregs population [17, 18]. A role of Bregs in hepatitis E virus infection has not been explored. The current study presents a comprehensive investigation of B regulatory cells in patients with hepatitis E infection. We have assessed (1) frequency and phenotypic markers of B regulatory cells and (2) functionality of B regulatory cells in acute hepatitis E patients, hepatitis E recovered individuals and healthy controls.

## 2. Materials and Methods

### 2.1. Patients and Controls

The study was approved by the Institutional Ethical Committee for Research on Humans, based on the guidelines set by the Indian Council of Medical Research (ICMR), New Delhi. Informed written consent was obtained from all the participants in accordance with the Declaration of Helsinki.

The study population (n =291) included the following groups: acute hepatitis E patients (n =108), hepatitis E recovered individuals (n =55) and anti-HEV negative healthy controls (n =128). The study groups were classified based on standard clinical and biochemical criteria [19]. Patient demographics are shown in Table 1.

*Inclusion criteria: Acute hepatitis E patients*: The patients were presenting with symptoms like icterus, dark coloured urine, fever, elevated alanine aminotransferase (ALT) levels (normal range 4-40 IU/L) and/or bilirubin >1 mg/mL in serum and/or presence of bile salts and pigments in urine. These patients were diagnosed positive for IgM antibodies against hepatitis E virus (anti-HEV IgM) and positive/negative for IgG antibodies against hepatitis E virus (anti-HEV IgG) by ELISA. Acute patients were from various outbreaks reported in India. *Hepatitis E recovered individuals*: The recovered category constituted of individuals with past history of acute hepatitis E infection and were from previously investigated hepatitis E outbreaks. These individuals were positive for anti-HEV IgG antibodies and negative for anti-HEV IgM antibodies with normal plasma ALT levels. The post onset days of illness for this group ranged from 0.2-2 years. *Healthy controls*: The control group constituted of age and sex matched apparently healthy individuals with no recent or past history of hepatitis E infection i.e. they were negative for anti-HEV IgM and IgG antibodies with normal ALT levels. The time of collection of healthy controls were same as that of the other two groups.

*Exclusion criteria*: Individuals with other viral infections including hepatitis A virus (HAV), HBV, HCV and HIV infections were excluded.

### 2.2. Serological and Molecular Assays

All samples were screened by in-house ELISA for anti-HEV IgM and IgG antibodies as described previously [20]. ALT levels were measured using GPT (ALT) test kit (Span Cogent Diagnostics, India) in all plasma samples as per manufacturer's protocol [21]. Plasma HEV viral load was determined by Taqman reverse transcription polymerase chain reaction as previously reported [22].

### 2.3. Antigen (Recombinant HEV-ORF2 Protein) Preparation

Recombinant ORF2 protein (rORF2p) was expressed in Sf9 insect cells and purified by anion exchange high performance liquid chromatography using AKTA BASIC 100 system (Amersham Biosciences, UK) as described previously [23]. rORF2p was used as the antigen for coating in ELISA for detection of anti-HEV IgM and IgG antibodies and as a recall antigen in the functional assays.

### 2.4. Peripheral Blood Mononuclear Cells (PBMCs) Isolation

PBMCs were isolated using Ficoll-Hypaque density gradient centrifugation method and counted in the trypan blue dye exclusion method and the cells with more than 98% of viability were immediately used for assays. Isolated plasma was used for serological and biochemical assays and the PBMCs were used for all assays. The number of samples from each category used for different assays is shown in Supplementary Figure S1.

### 2.5. Frequencies of HEV-rORF2p Stimulated B Cells and Its Subsets

PBMCs from 40 acute hepatitis E patients, 29 hepatitis E recovered individuals and 50 healthy controls were cultured in complete Roswell Park Memorial Institute (RPMI) 1640 medium i.e. RPMI-1640+10% FBS (Gibco, USA) with/without HEV-rORF2p (10 *μ*g/mL) for 72 h. The PBMCs from representative samples from each category of study subjects were also incubated with inactivated chikungunya virus (CHIKV, 10 *μ*g/mL) as a non-specific stimulation protein for 72 h and the frequencies of B cells and its subsets were determined. Cells were harvested and 1 × 10^6^ unstimulated/stimulated cells were surface stained using anti-CD19 PerCp-Cy5.5, anti-CD24 PE and anti-CD38 FITC antibodies (BD Biosciences, CA, USA). For each sample, 50,000 events were acquired in BD FACS Aria-II flow cytometer and analyzed using BD FACS Diva software (BD Biosciences, CA, USA). The strategy for gating B, immature B, mature B and memory B cells is depicted in (Supplementary Figure S2). B cells (CD19^+^) were gated from lymphocytes while immature/transitional B (CD19^+^CD24^hi^CD38^hi^), mature B (CD19^+^CD24^int^CD38^int^) and memory B (CD19^+^CD24^hi^CD38^−^) cells were gated from their parent cells. Data from HEV-rORF2p stimulated cells were analyzed after normalization with that of unstimulated cells for each sample.

### 2.6. Assessment of IL-10 and TGF-*β* Cytokines Expression by Intracellular Cytokine Staining

The expression of cytokines IL-10 and TGF-*β* by B cells and its subsets were assessed by intracellular cytokine staining. PBMCs from 36 acute hepatitis E patients, 29 hepatitis E recovered individuals and 51 healthy controls were incubated with 1 *μ*M CpG-B ODN2006 (InvivoGen, CA, USA) for 72 h at 37°C. Phorbol myristate acetate (PMA) (25 ng/mL) and ionomycin (1 *μ*g/mL) (Sigma, USA) were added in the last 5 h in the presence of 10 *μ*g/mL Brefeldin-A (Sigma, USA). Alternatively, PBMCs were stimulated with HEV-rORF2p (10 *μ*g/mL) for 72 h. Cells were then surface stained for the markers CD19 PE-Cy7, CD24 PE and CD38 FITC (BD Biosciences, CA, USA), fixed, permeabilized, and stained intracellularly with anti-IL-10 BV421 and TGF-*β* PerCP-Cy5.5 antibodies (BD Biosciences, CA, USA). Isotype and Fluorescence Minus One (FMO) controls were used in all sets of experiments. For each sample, 50,000 events were acquired in BD FACS Aria-II flow cytometer and data were analyzed using BD FACS Diva software (BD Biosciences, CA, USA). Data from stimulated cells were analyzed after normalization with unstimulated cells. The gating strategy is depicted in (Supplementary Figure S2).

### 2.7. Quantification of IL-10 Cytokine in Plasma Samples

Plasma concentrations of IL-10 cytokine were determined in representative plasma samples from each category of study subjects (acute hepatitis E patients, n =20; hepatitis E recovered individuals, n =20 and healthy controls, n =27) using a Bio-plex Multiplex Immunoassay System (Bio-Rad, Hercules, CA, USA) using a Bioplex ProTM Human Cytokine 27-plex assay kit as reported previously [24, 25] as per the manufacturer's instructions.

### 2.8. Assessment of HEV-rORF2p Specific T Cell Responses Pre/Post IL-10/IL-10R Blocking

The effect of blocking of IL-10 and IL-10 receptor (IL-10R) on T cell responses was assessed by blocking assay. PBMCs from 46 acute hepatitis E patients, 23 hepatitis E recovered individuals and 54 healthy controls were cultured in 24-well plate (0.3 × 10^6^ cells/well) in the presence/absence of HEV-rORF2p (10 *μ*g/mL) and 50 U/mL IL-2 (BD Biosciences, CA, USA) with or without anti-IL10 (5 *μ*g/mL; BD Biosciences, CA, USA) and anti-IL10R (10 *μ*g/mL; BD Biosciences, CA, USA) antibodies and incubated at 37°C for 10 days. On day 4 and 7, medium was changed followed by fresh addition of recombinant IL-2 (50 U/mL), anti-IL10 (5 *μ*g/mL) and anti-IL10R (10 *μ*g/mL). At the end of 10 days incubation, brefeldin A (10 *μ*g/mL) was added in the last 5 h and PBMCs were stained with anti-CD3 APC-H7, anti-CD4 PE-Cy7 and anti-CD8 V450 antibodies followed by intracellular staining with IFN-*γ* Alexa Fluor 647 antibody (BD Biosciences, CA, USA). HEV-rORF2p specific IFN-*γ* expression on CD4 and CD8 T cells was assessed by flow cytometry. Isotype and FMO controls were used in all sets of experiments. The gating strategy is shown in (Supplementary Figure S3).

### 2.9. CD19^+^IL-10^+^ Cells Depletion-Based B Cell Functional Assay

To determine functionality of IL-10 expressing B cells, PBMCs from 33 acute hepatitis E patients, 22 hepatitis E recovered individuals and 52 healthy controls were processed for isolation of B cells. B cells were positively enriched from PBMCs using human CD19 MicroBeads (Miltenyi Biotec, CA, USA) and were separated over MACS MS Column (Miltenyi Biotec, CA, USA). The purity of the sorted population was 95–99%, as determined by flowcytometry. Isolated CD19^+^ and CD19^−^ cells were stimulated with/without HEV-rORF2p (10 *μ*g/mL) for 24 h at 37°C followed by detection and isolation of IL-10 expressing B cells using human IL-10 cytokine secretion assay (Miltenyi Biotec, CA, USA) as per manufacturer's protocol. Further, magnetically sorted CD19^+^IL-10^+^ and CD19^−^IL-10^−^ cells were stimulated with/without HEV-rORF2p (10 *μ*g/mL) and co-cultured with PBMCs derived from the same patients/controls at 1 : 1 ratio in round bottom 96-well plate (Nunc, Denmark) in the presence of recombinant IL-2 (50 IU/mL) for 5 days. This was followed by intracellular staining with IFN-*γ* Alexa Fluor 647 antibody (BD Biosciences, CA, USA). The frequencies of HEV-rORF2p specific IFN-*γ* expression on CD4 and CD8 T cells in the presence and absence of IL-10^+^ B cells was determined. Isotype and FMO controls were used in all sets of experiments. The gating strategy is shown in (Supplementary Figure S3).

### 2.10. Statistical Analysis

SPSS 20 software (SPSS Inc., IL, USA) was used for all the statistical analyses. Statistical significance was calculated by the nonparametric Mann–Whitney U-test (where difference in variances <4) or Kolmogorov–Smirnov test (where difference in variances >4). Pair wise comparisons were done using the Wilcoxon's signed-rank test on matched samples. A p value of <0.05 was deemed significant. The data are expressed as median (range). Correlation among HEV viral load, ALT levels, anti-HEV IgM/IgG titers, plasma IL-10 cytokine concentrations and HEV-rORF2p specific B cells and its subsets frequencies was assessed using Spearman's rank correlation. Only significant results are presented.

## 3. Results

### 3.1. HEV-rORF2p Stimulated B Cells and Its Subsets Frequencies

**Figure 1:**
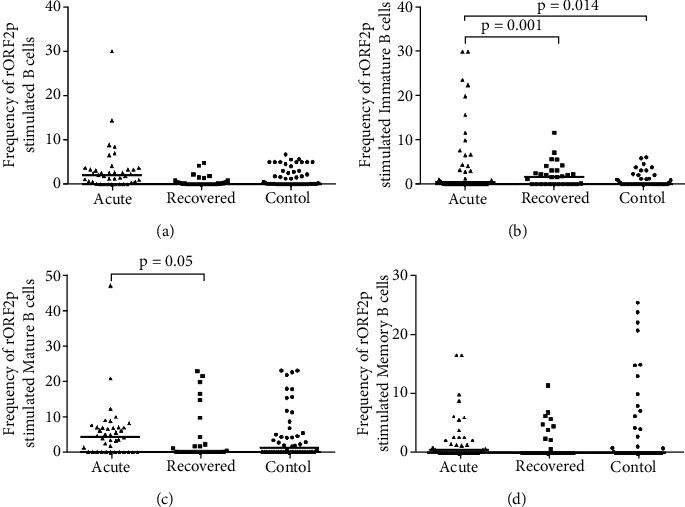
Flow cytometric analysis of B, immature B, mature B and memory B cells upon stimulation with recombinant HEV open reading frame 2 protein (HEV-rORF2p). PBMCs from 40 acute hepatitis E patients, 29 hepatitis E recovered individuals, and 50 healthy controls were cultured in the presence/absence of HEV-rORF2p. After 72 h, cells were harvested, stained and acquired on flow cytometer. Frequencies of HEV-rORF2p stimulated (a) B, (b) immature B, (c) mature B and (d) memory B cells were determined after normalization with unstimulated cells. B cells (CD19^+^) were gated from lymphocytes while immature/transitional B (CD19^+^CD24^hi^CD38^hi^), mature B (CD19^+^CD24^int^CD38^int^) and memory B (CD19^+^CD24^hi^CD38^−^) cells were gated from their parent cells. The dots represent individual values and bars represent median values.

In order to examine the phenotype of Bregs post HEV-rORF2p stimulation, percentages of B cells and its subsets were enumerated in acute hepatitis E patients, hepatitis E recovered individuals and healthy controls by flow cytometry. The frequencies of B cells were comparable among all the study groups [acute: 2 (0–29.8), recovered: 0.1 (0–4.8), controls: 0.2 (0–6.7); acute vs. controls (p =0.098), acute vs. recovered (p =0.106), recovered vs. controls (p =0.206)] (Figure 1(a)). A significantly higher frequencies of immature B cells were observed in acute hepatitis E patients compared to healthy controls [acute: 0.5 (0–29.7) vs. controls: 0 (0–6), p =0.014] and hepatitis E recovered individuals [acute: 0.5 (0–29.7) vs. recovered: 0 (0–11.5), p =0.001] (Figure 1(b)). Mature B cell frequencies were comparable among acute hepatitis E patients and hepatitis E recovered individuals compared to healthy controls [acute: 4.3 (0–47), recovered: 0 (0–22.9), controls: 1.2 (0–23.1); acute vs. controls (p =0.156), recovered vs. controls (p =0.136)], whereas among the patient categories, mature B cells frequencies were higher in acute hepatitis E patients [acute: 4.3 (0–47) vs. recovered: 0 (0–22.9), p =0.05] (Figure 1(C)). Further, the frequencies of memory B cells were comparable among all the study groups [acute: 0.5 (0–16.4), recovered: 0 (0–11.3), controls: 0 (0–25.2); acute vs. controls (p =0.272), acute vs. recovered (p =0.208), recovered vs. controls (p =0.198)] (Figure 1(D)). The frequencies of B cells and its subsets upon CHIKV stimulation were comparable among all the study groups [B cells: acute vs. controls (p =0.153), acute vs. recovered (p =0.108), recovered vs. controls (p =0.098); Immature B cells: acute vs. controls (p =0.953), acute vs. recovered (p =0.446), recovered vs. controls (p =0.081); Mature B cells: acute vs. controls (p =0.263), acute vs. recovered (p =0.318), recovered vs. controls (p =0.775); Memory B cells: acute vs. controls (p =0.235), acute vs. recovered (p =0.82), recovered vs. controls (p =0.208)].

### 3.2. Functional Analysis of B Regulatory Cells

#### 3.2.1. Expression of IL-10 and TGF-*β* on B Cells and Its Subsets during Hepatitis E Infection and following Recovery

We next analysed the frequencies of IL-10 and TGF-*β* expressing B cells and its subsets in acute hepatitis E patients, hepatitis E recovered individuals and healthy controls by intracellular cytokine staining.

**Figure 2:**
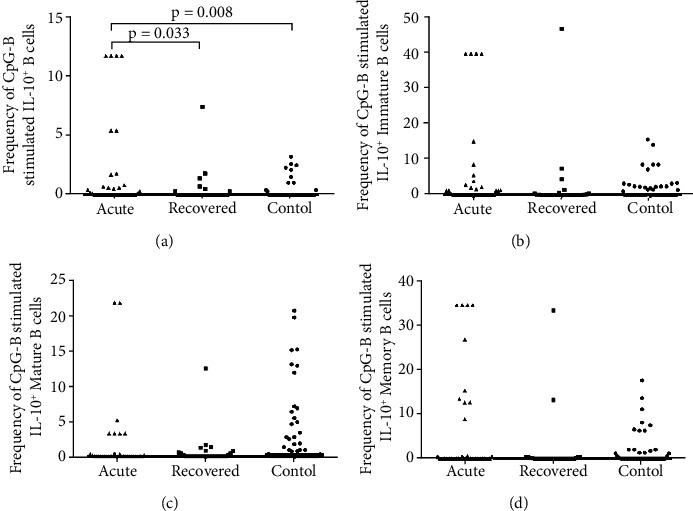
Intracellular cytokine staining for IL-10 upon CpG-B stimulation. Expression of IL-10 on (a) B, (b) immature B, (c) mature B and (d) memory B cells upon CpG-B stimulation were determined in 36 acute hepatitis E patients, 29 hepatitis E recovered individuals, and 51 healthy controls. The dots represent individual values and bars represent median values.

*(1) Expression of IL-10 in Response to CpG-B Stimulus*. In response to CpG-B stimulus, it was observed that the frequencies of IL-10^+^ B cells were higher in acute hepatitis E patients compared to healthy controls [acute: 0.05 (0–11.7) vs. controls: 0 (0–3.2), p =0.008] and hepatitis E recovered individuals [acute: 0.05 (0–11.7) vs. recovered: 0 (0–7.4), p =0.033] (Figure 2(a)). The frequencies of IL-10^+^ immature B cells [acute: 0.15 (0–39.7), recovered: 0 (0–46.7), controls: 0 (0–15.6); acute vs. controls (p =0.481), acute vs. recovered (p =0.102), recovered vs. controls (p =0.148)] (Figure 2(b)), IL-10^+^ mature B cells [acute: 0 (0–21.7), recovered: 0 (0–12.4), controls: 0.3 (0–20.6); acute vs. controls (p =0.074), acute vs. recovered (p =0.865), recovered vs. controls (p =0.446)] (Figure 2(C)) and IL-10^+^ memory B cells [acute: 0 (0–34.5), recovered: 0 (0–33.3), controls: 0.3 (0–17.6); acute vs. controls (p =0.662), acute vs. recovered (p =0.179), recovered vs. controls (p =0.369)] (Figure 2(D)) were comparable among all study groups.

**Figure 3:**
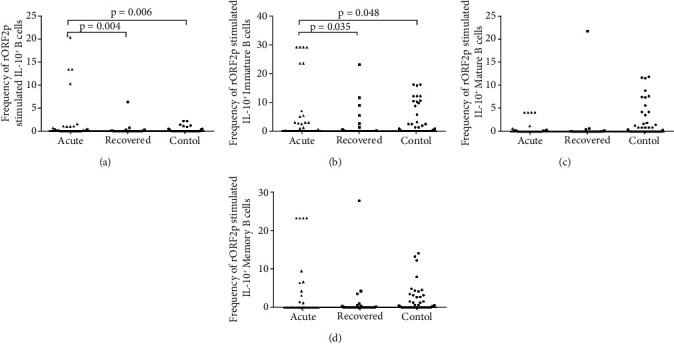
Intracellular cytokine staining for IL-10 post HEV-rORF2p stimulation. Expression of IL-10 on (a) B, (b) immature B, (c) mature B and (d) memory B cells post HEV-rORF2p stimulation were determined in 36 acute hepatitis E patients, 29 hepatitis E recovered individuals, and 51 healthy controls. The dots represent individual values and bars represent median values.

*(2) Expression of IL-10 in Response to HEV-rORF2p Antigen*. The frequencies of IL-10^+^ B cells were higher in acute hepatitis E patients compared to healthy controls [acute: 0.15 (0-20.3) vs. controls: 0 (0–2.2), p =0.006] and hepatitis E recovered individuals [acute: 0.15 (0–20.3) vs. recovered: 0 (0–6.3), p =0.004] (Figure 3(a)) in response to HEV-rORF2p antigen stimulation. A significant elevation in the frequencies of IL-10^+^ immature B cells was observed in acute hepatitis E patients compared to healthy controls [acute: 0.2 (0–28.9) vs. controls: 0 (0–16), p =0.048] and hepatitis E recovered individuals [acute: 0.2 (0–28.9) vs. recovered: 0 (0–22.9), p =0.035] (Figure 3(b)). The frequencies of IL-10^+^ mature B cells [acute: 0 (0–4.2), recovered: 0 (0–21.8), controls: 0 (0–11.9); acute vs. controls (p =0.281), acute vs. recovered (p =0.085), recovered vs. controls (p =0.179)] (Figure 3(C)) and IL-10^+^ memory B cells [acute: 0 (0–23.3), recovered: 0 (0–27.8), controls: 0.1 (0–14.2); acute vs. controls (p =0.382), acute vs. recovered (p =0.525), recovered vs. controls (p =0.366)] (Figure 3(D)) were comparable among the study groups.

**Figure 4:**
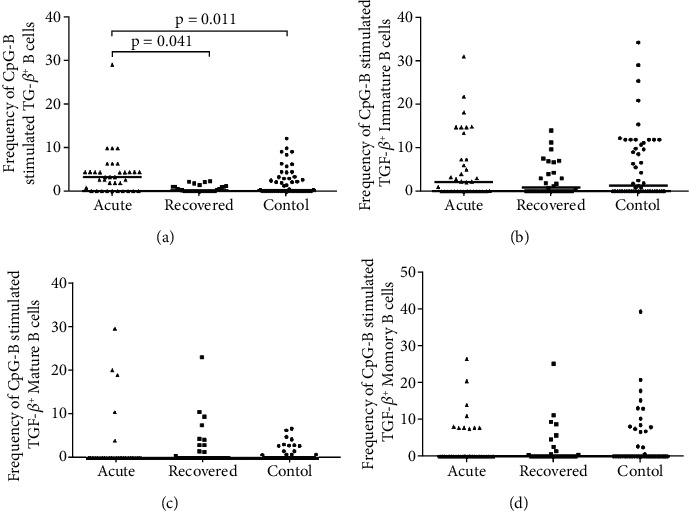
Intracellular cytokine staining for TGF-*β* post CpG-B stimulation. Expression of TGF-*β* on (a) B, (b) immature B, (c) mature B and (d) memory B cells post CpG-B stimulation were determined in 36 acute hepatitis E patients, 29 hepatitis E recovered individuals, and 51 healthy controls. The dots represent individual values and bars represent median values.

*(3) Expression of TGF-β in Response to CpG-B Stimulus*. In response to CpG-B stimulus, the frequencies of TGF-*β*^+^ B cells were higher in acute hepatitis E patients compared to healthy controls [acute: 1.9 (0–29) vs. controls: 0.3 (0–12.1), p =0.011] and hepatitis E recovered individuals [acute: 1.9 (0–29) vs. recovered: 0.2 (0–2.3), p =0.041] (Figure 4(a)). The frequencies of TGF-*β*^+^ immature B cells [acute: 1.8 (0–30.9), recovered: 0.9 (0–13.9), controls: 1.3 (0–34); acute vs. controls (p =0.371), acute vs. recovered (p =0.401), recovered vs. controls (p =0.439)] (Figure 4(b)), TGF-*β*^+^ mature B cells [acute: 0 (0–29.7), recovered: 0 (0–23.1), controls: 0 (0–6.9); acute vs. controls (p =0.296), acute vs. recovered (p =0.093), recovered vs. controls (p =0.233)] (Figure 4(C)) and TGF-*β*^+^ memory B cells [acute: 0 (0–26.3), recovered: 0 (0–25), controls: 0 (0–39.1); acute vs. controls (p =0.709), acute vs. recovered (p =0.821), recovered vs. controls (p =0.645)] (Figure 4(D)) were comparable among the study groups.

**Figure 5:**
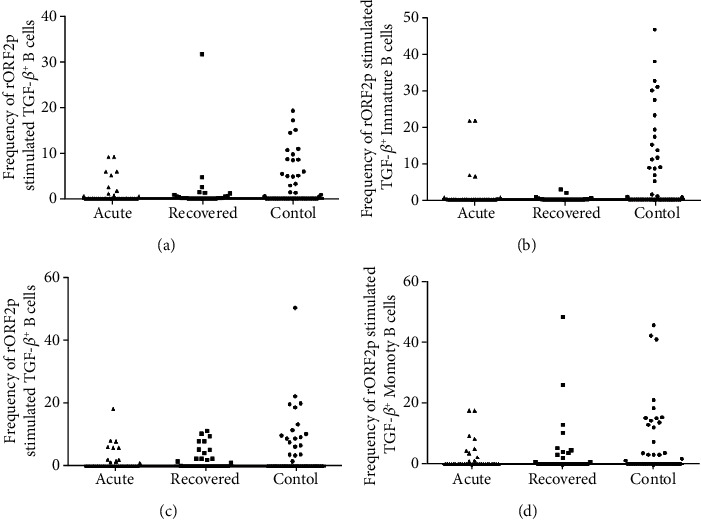
Intracellular cytokine staining for TGF-*β* post HEV-rORF2p stimulation. Expression of TGF-*β* upon HEV-rORF2p stimulation on (a) B, (b) immature B, (c) mature B and (d) memory B cells were determined in 36 acute hepatitis E patients, 29 hepatitis E recovered individuals, and 51 healthy controls. The dots represent individual values and bars represent median values.

*(4) Expression of TGF-β in Response to HEV-rORF2p Antigen*. The frequencies of TGF-*β*^+^ B cells were comparable among the study groups post HEV-rORF2p antigen stimulation [acute: 0 (0–9.1), recovered: 0.1 (0–31.6), controls: 0 (0–19.2); acute vs. controls (p =0.169), acute vs. recovered (p =0.249), recovered vs. controls (p = 0.28)] (Figure 5(a)). Similarly, the frequencies of TGF-*β*^+^ immature B cells [acute: 0 (0–21.6), recovered: 0 (0–2.8), controls: 0 (0–46.6); acute vs. controls (p = 0.133), acute vs. recovered (p = 0.741), recovered vs. controls (p = 0.601)] (Figure 5(b)), TGF-*β*^+^ mature B cells [acute: 0 (0–18.2), recovered: 0.2 (0–11.2), controls: 0 (0–50.3); acute vs. controls (p = 0.512), acute vs. recovered (p = 0.302), recovered vs. controls (p =0.179)] (Figure 5(C)) and TGF-*β*^+^ memory B cells [acute: 0 (0–17.6), recovered: 0 (0–48.4), controls: 0 (0–45.7); acute vs. controls (p = 0.324), acute vs. recovered (p = 0.605), recovered vs. controls (p = 0.755)] (Figure 5(D)) were also found to be comparable among the study groups.

The above results collectively suggest that Bregs are functional and IL-10 was noted to be the predominant cytokine expressed on Bregs in acute HEV infection.

#### 3.2.2. Correlation between Plasma IL-10 Levels and Immature B Cells Frequencies

**Figure 6:**
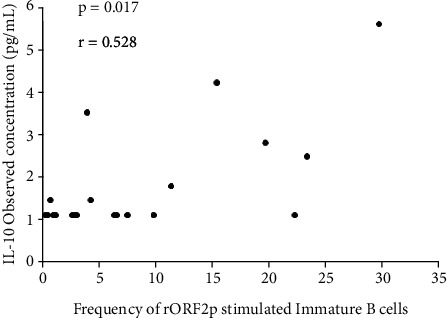
Correlation between plasma IL-10 levels and immature B cells frequencies. Spearman correlation co-efficient shows positive correlation between plasma IL-10 concentrations of the acute hepatitis E patients and HEV-rORF2p stimulated immature B cells frequencies, where p < 0.05 is considered significant and r > 0.5 is considered as positive.

A positive correlation was observed between plasma IL-10 concentrations of the acute hepatitis E patients and HEV-rORF2p stimulated immature B cells frequencies (r = 0.528, p = 0.017) (Figure 6).

#### 3.2.3. Assessment of HEV-rORF2p Specific T Cell Responses (in terms of IFN-*γ* Expression) Pre and Post IL-10/IL-10 Receptor Blocking during Hepatitis E Infection and following Recovery

**Figure 7:**
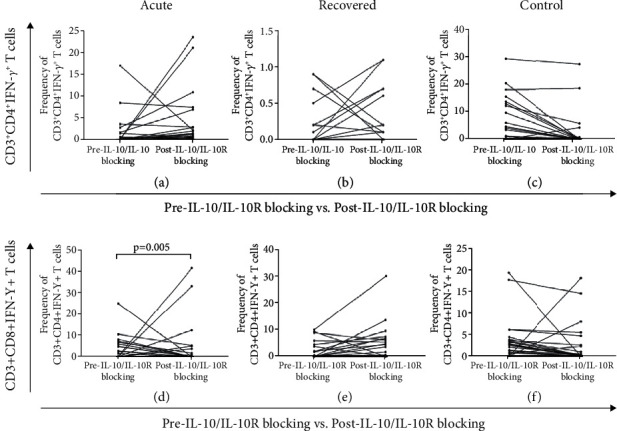
Flow cytometric analysis showing the effect of IL-10/IL-10R blocking on HEV-rORF2p specific CD4 and CD8 T cells responses. PBMCs from 46 acute hepatitis E patients, 23 hepatitis E recovered individuals, and 54 healthy controls stimulated in the presence/absence of HEV-rORF2p, were cultured with/without IL-10/IL-10R blocking antibodies in the presence of IL-2. On tenth day, the cells were intracellularly stained to determine expression of IFN-*γ* on CD4^+^ T cells (%CD3^+^CD4^+^IFN-*γ*^+^) and CD8^+^ T cells (%CD3^+^CD8^+^IFN-*γ*^+^). Frequencies of CD3^+^CD4^+^IFN-*γ*^+^ T cells in (a) acute hepatitis E patients, (b) hepatitis E recovered individuals, (c) healthy controls and CD3^+^CD8^+^IFN-*γ*^+^ T cells in (d) acute hepatitis E patients, (e) hepatitis E recovered individuals, (f) healthy controls were assessed pre vs. post IL-10/IL-10R blocking. Pair wise comparisons were done on matched samples wherein dots represent individual values.

Effect of IL-10/IL-10R blocking on HEV rORF2p specific CD4 and CD8 T cell responses was assessed by *in-vitro* IL-10/IL-10R blocking assay. The frequencies of HEV-rORF2p specific IFN-*γ* expression on CD4^+^ T cells (%CD3^+^CD4^+^IFN-*γ*^+^) were comparable among the study groups pre and post IL-10/IL-10R blocking [pre IL-10/IL-10R blocking - acute: 0 (0-17), recovered: 0 (0-0.9), controls: 0 (0-29.8) vs. post IL-10/IL-10R blocking - acute: 0.35 (0-23.5), recovered: 0.1 (0-1.1), controls: 0 (0-27.6); acute (p = 0.376), recovered (p = 0.483), controls (p =0.074)] (Figures 7(a), 7(b), 7(C)). However, the frequencies of HEV-rORF2p specific IFN-*γ* expression on CD8^+^ T cells (%CD3^+^CD8^+^IFN-*γ*^+^) were higher in acute hepatitis E patients post IL-10/IL-10R blocking [pre IL-10/IL-10R blocking – acute: 0 (0–25) vs. post IL-10/IL-10R blocking – acute: 0.65 (0–41.7), p = 0.005] (Figure 7(D)). The frequencies of HEV-rORF2p specific IFN-*γ* expression on CD8^+^ T cells pre and post IL-10/IL-10R blocking were comparable in hepatitis E recovered individuals and in healthy controls [pre IL-10/IL-10R blocking - recovered: 0 (0-10.1), controls: 0 (0-19.4) vs. post IL-10/IL-10R blocking – recovered: 1.7 (0-30.2), controls: 0 (0-18.2); recovered (p = 0.065), controls (p =0.085)] (Figures 7(e), 7(f)).

#### 3.2.4. Assessment of HEV Specific T Cell Responses Pre and Post CD19^+^IL-10^+^ Cells Depletion

The effect of CD19^+^IL-10^+^ B cells on HEV-rORF2p specific CD4 and CD8 T cells responses was assessed in all study subjects before and after depletion of CD19^+^IL-10^+^ cells. The expression of IFN-*γ* on HEV-rORF2p specific CD4 T cells (%CD3^+^CD4^+^IFN-*γ*^+^) was comparable among the study groups, pre and post depletion of CD19^+^IL-10^+^ B cells [pre CD19^+^IL-10^+^ B cells depletion - acute: 0 (0–72.4), recovered: 0 (0-28.2), controls: 0.55 (0-37.7) vs. post CD19^+^IL-10^+^ B cells depletion - acute: 0 (0–48.9), recovered 0.3 (0-3.6), controls 0.8 (0-18.6); acute (p =0.198), recovered (p = 0.348), controls (p = 0.53)] (Figures Supplementary Materials (a), Supplementary Materials (b), Supplementary Materials (c)). However, the expression of IFN-*γ* on HEV-rORF2p specific CD8 T cells (CD3^+^CD8^+^IFN-*γ*^+^) cells was higher in acute hepatitis E patients post depletion of CD19^+^IL-10^+^ B cells [pre CD19^+^IL-10^+^ B cells depletion - acute: 0 (0–20.3) vs. post CD19^+^IL-10^+^ B cells depletion - acute: 0.4 (0–35.7), p = 0.021] (Figure Supplementary Materials(d)). Depletion of CD19^+^IL-10^+^ B cells did not have any effect in hepatitis E recovered individuals and healthy controls [pre CD19^+^IL-10^+^ B cells depletion - recovered: 0 (0-11.5), controls: 0 (0-3.4) vs. post CD19^+^IL-10^+^ B cells depletion - recovered 0.15 (0-9.9), controls 0 (0-0.3); recovered (p = 0.293), controls (p = 0.138)] (Figures Supplementary Materials(e), Supplementary Materials(f)).

### 3.3. Lack of Correlation between HEV Viral Load, ALT Levels, anti-HEV IgM/IgG Titers and HEV-rORF2p Stimulated B Cells and Its Subsets Frequencies in Acute Hepatitis E Patients

There was no correlation between HEV viral load, ALT levels, anti-HEV IgM/IgG titers and HEV-rORF2p stimulated B, immature B, mature B and memory B cells frequencies in acute hepatitis E patients as assessed by spearman correlation analysis. Upon assessing the role of viral replication with the IL-10 producing B cells, it was observed that only a non-significant proportion of viremic acute hepatitis E patients had IL-10 producing B cells (17/36, 47.22%), further suggesting no association of viral replication with Bregs functionality in the self-limiting acute hepatitis E patients.

## 4. Discussion

The present study is an in-depth assessment of the role of B regulatory cells in self-limiting HEV infection where the immunoregulatory role of Bregs is being presented.

The percentages of B cells remained unaltered among our study cohort which goes in parallel with a study from our group evaluating role of B and memory B cells in HEV infection [26]. Similar observation was noted by Das et al. in a cohort of chronic hepatitis B patients [4]. Significant augmentation of HEV-rORF2p specific immature CD19^+^CD24^hi^CD38^hi^ B cells phenotype in self-limiting acute hepatitis E patients compared to hepatitis E recovered individuals and healthy controls of our study mirrors similar observations documented in HBV [4], HIV and hepatitis C virus (HCV) infections [27, 28]. Freshly isolated human IL-10^+^ Bregs have predominantly been detected within the CD24 or CD27 B cell subpopulations; Bregs were subsequently characterized as memory CD19^+^CD24^hi^CD27^+^ B cells by Tedder [29]. The discrepancies observed in phenotypes of Bregs between studies could partially be due to absence of a perfect panel of markers for characterization of this subset of B cells. However, though different phenotypes of human Bregs have been reported in different disease settings, only certain B cells have been shown to produce IL-10 [14, 30].

Enriched HEV-rORF2p stimulated immature B cells in acute hepatitis E patients compared to both recovered individuals and controls, in spite of having comparable HEV-rORF2p stimulated CD19^+^ B cells among the study population is suggestive of the theory that a subset of B cells could have expanded from the existing pool of B cells. This is also suggestive of second model of Bregs development and differentiation pointing towards differentiation and expansion of this subset in response to external stimulation, HEV-rORF2p antigen in this case [31]. Existence of enhanced HEV antigen specific immature CD19^+^CD24^hi^CD38^hi^ B cells and mature CD19^+^CD24^int^CD38^int^ B cells in acute hepatitis E patients' group compared to recovered group may support heterogeneous existence of phenotypes of Bregs [31].

We chose to study immature B cells as the putative regulatory subset in HEV infection. IL-10 production has remained the hallmark of Bregs regulatory function, though TGF-*β*, IL-35 and IL-17 mediated several regulatory mechanisms have also come to light in recent years [32]. Simultaneous expressions of regulatory cytokines like IL-10 and TGF-*β* on Bregs have been demonstrated [33]. IL-10 is known to have a prognostic/detrimental role in infections [34]. Significantly higher IL-10 expression on CD19^+^ B and HEV-rORF2p stimulated CD19^+^ immature B cells of acute hepatitis E patients of the current study indicated that Bregs are functional. Further, a positive correlation was also observed between plasma IL-10 concentrations and HEV-ORF2p stimulated immature B cells frequencies in acute hepatitis E patients. We next demonstrated that IL-10^+^ B cells were of immature B cells (CD19^+^CD24^hi^CD38^hi^) phenotype only. It is important to note that though frequencies of HEV-rOFR2p stimulated mature B cells were more in acute hepatitis E patients compared to hepatitis E recovered individuals their functionality was comparable as indicated by the comparable frequencies of IL-10^+^ mature B cells. Interestingly, IL-10 was noted to be the predominant cytokine expressed on Bregs in acute HEV infection. Similarly, elevated levels of IL-10 producing B cells were observed in patients with HIV, HCV and lymphocytic choriomeningitis virus (LCMV) infections and the increased IL-10 levels were correlated with suppression of T cells [35]. Different methods of stimulation could impact the induction of IL-10 production by Bregs [9]. Upon stimulation with CpG-B, an agonist of TLR-9, we observed an induction of TGF-*β* expression on B cells and these TGF-*β*^+^ B cells were enriched in acute hepatitis E patients compared to recovered individuals and healthy controls. However, there was no detection of HEV-rORF2p specific TGF-*β* expression on B cells or B cells subsets which explain that most of the primary functions of Bregs cells in HEV infection are dependent on expression and release of IL-10. It is quite reasonable that Bregs act as the main source of IL-10 and may be considered as an immune regulator in self-limiting acute HEV infection.

HEV RNA positivity in sera of hepatitis E patients lasts beyond normalization of transaminases suggesting that liver injury is independent of viral replication [36]. We attempted to associate viremia and liver damage with the functionality of Bregs in acute hepatitis E patients. No correlation of increased ALT levels and viral replication with IL-10 producing B cells indicated that functional Bregs have probably no impact on the liver damage of the acute hepatitis E patients.

Bregs mediated CD8^+^ T cells inhibition and suppression of inflammation has been extensively studied in infectious diseases and cancers [32]. Das et al. have documented that sorted CD19^+^CD24^hi^CD38^hi^ cells suppressed HBV-specific CD8 T cell responses in an IL-10 dependent manner in chronic HBV infection [4]. A significant increase in IFN-*γ* expression on CD8^+^ T cells and no effect on IFN-*γ* expression on CD4^+^ T cells upon blocking of both IL-10 and IL-10R and depletion of CD19^+^IL-10^+^ B cells in our study is an important observation. Current data clearly indicate that Bregs in Hepatitis E mediate T cell modulation via CD8^+^ T cells.

It could be suggested that CD3^+^CD8^+^IFN-*γ*^+^ T cells in acute hepatitis E patients corroborated the regulatory potential of Bregs via IL-10 dependent mechanism. These observations of our study are partly in concordance as reported in HIV infection where Bregs suppress HIV-1 specific CD8^+^ T-cell responses via IL-10 production and possibly PD-L1 expression [37]. In the past, we have proposed a beneficial role for Tregs in self-limiting acute HEV infection based on the higher percentage of HEV specific, functional Tregs in self-limiting HEV patients [15, 16, 38] and absence of the Tregs in the liver of fatal hepatitis E FHF patients [39]. We had further elucidated TGF-*β*1 as the regulatory molecule responsible for enhancement of Tregs in self-limiting HEV infection and use of TGF-*β*1 was suggested as a possible supplement for boosting Treg response towards recovery from severe hepatitis E [16]. IL-10 producing B cells are known to play a major role towards the induction and maintenance of Tregs [9]. Our current results definitely suggest participation of Bregs in the maintenance of immune homeostasis and may allude possible existence of Bregs-Tregs interactions in HEV disease pathogenesis. It is tempting to speculate the mechanism of interaction among these two groups of regulatory cells.

The limitation of this study was that the role of Bregs was not explored among FHF patients with differential outcomes. Indeed, sans Bregs data of FHF patients, the prognostic/pathogenic role of Bregs in HEV infection remains an avenue unexplored. In the absence of any information about the composition of B cells among PBMCs before stimulation, the answers to questions including the differences due to composition of B cells before and after stimulation, cell survival/cell proliferation/effect of Bregs on induction of B cell populations and antibody generation are beyond the scope of the current dataset and can be considered as a limitation of the study.

In a nutshell, we have identified HEV specific functional, immature CD19^+^CD24^hi^CD38^hi^ B cells having IL-10 mediated regulatory activities that have the potential to modulate IFN-*γ* mediated T cell response in Hepatitis E infection.

## Figures and Tables

**Figure 8:**
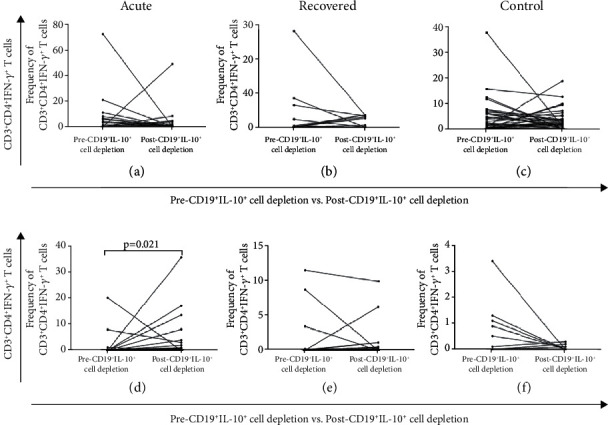
Flow cytometric analysis showing the effect of depletion of CD19^+^IL-10^+^ cells on HEV-rORF2p specific CD4 and CD8 T cells response in terms of IFN-*γ* expression. B cells were magnetically sorted from PBMCs from 33 acute hepatitis E patients, 22 hepatitis E recovered individuals and 52 healthy controls, and were stimulated with/without HEV-rORF2p followed by detection and isolation of IL-10 expressing B cells. Sorted CD19^+^IL-10^+^ and CD19^−^IL-10^−^ cells were co-cultured with PBMCs derived from the same patients/controls and were intracellularly stained to determine the expression of IFN-*γ* on CD4^+^ T cells (%CD3^+^CD4^+^IFN- *γ*^+^) and CD8^+^ T cells (%CD3^+^CD8^+^IFN- *γ*^+^). Expression of IFN-*γ* on HEV-rORF2p specific CD4 T cells (CD3^+^CD4^+^IFN-*γ*^+^) in (a) acute hepatitis E patients, (b) hepatitis E recovered individuals, (c) healthy controls and CD8 T cells (CD3^+^CD8^+^IFN-*γ*^+^) in (d) acute hepatitis E patients, (e) hepatitis E recovered individuals, (f) healthy controls were assessed pre vs. post CD19^+^IL-10^+^ cells depletion. Pair wise comparisons were done on matched samples wherein dots represent individual values.

**Table 1:** Characteristics of study population.

Parameters	Acute hepatitis E patients (n =108)	Hepatitis E recovered individuals (n =55)	Healthy controls (n =128)
Age (in years)	30 (18-65)	39 (20-63)	28 (20-78)
POD	4 (1-18) days	2 (0.2-12) years	NA
ALT (IU/L)	90 (42-390)	12 (1-35)	8 (1-26)
Anti-HEV IgM	Positive	Negative	Negative
IgM titers	3200 (100-409600)	NA	NA
Anti-HEV IgG	Positive	Positive	Negative
IgG titers	6400 (100-409600)	12800 (100-25600)	NA
Male:Female ratio	89 : 19	31 : 24	91 : 37

All data are expressed as median (range). POD: Post onset days of illness. NA: Not applicable.

## Data Availability

The data used to support the findings of this study are included within the supplementary information file.

## Supplementary Materials

Supplementary Materials The number of samples from each category and the gating strategies used for different assays are presented as supplementary information.
