# Supplementary material for: Regulatory Role of B Cells and Its Subsets in Hepatitis E Virus Infection
Source: Biomed Res Int. 2022 Sep 12;2022:7932150. doi: 10.1155/2022/7932150 (PMC9484887; doi:10.1155/2022/7932150)
Supplement: Supplementary Materials — The number of samples from each category and the gating strategies used for different assays are presented as supplementary information. [file 7932150.f1.docx]

**Supplementary information:**

**
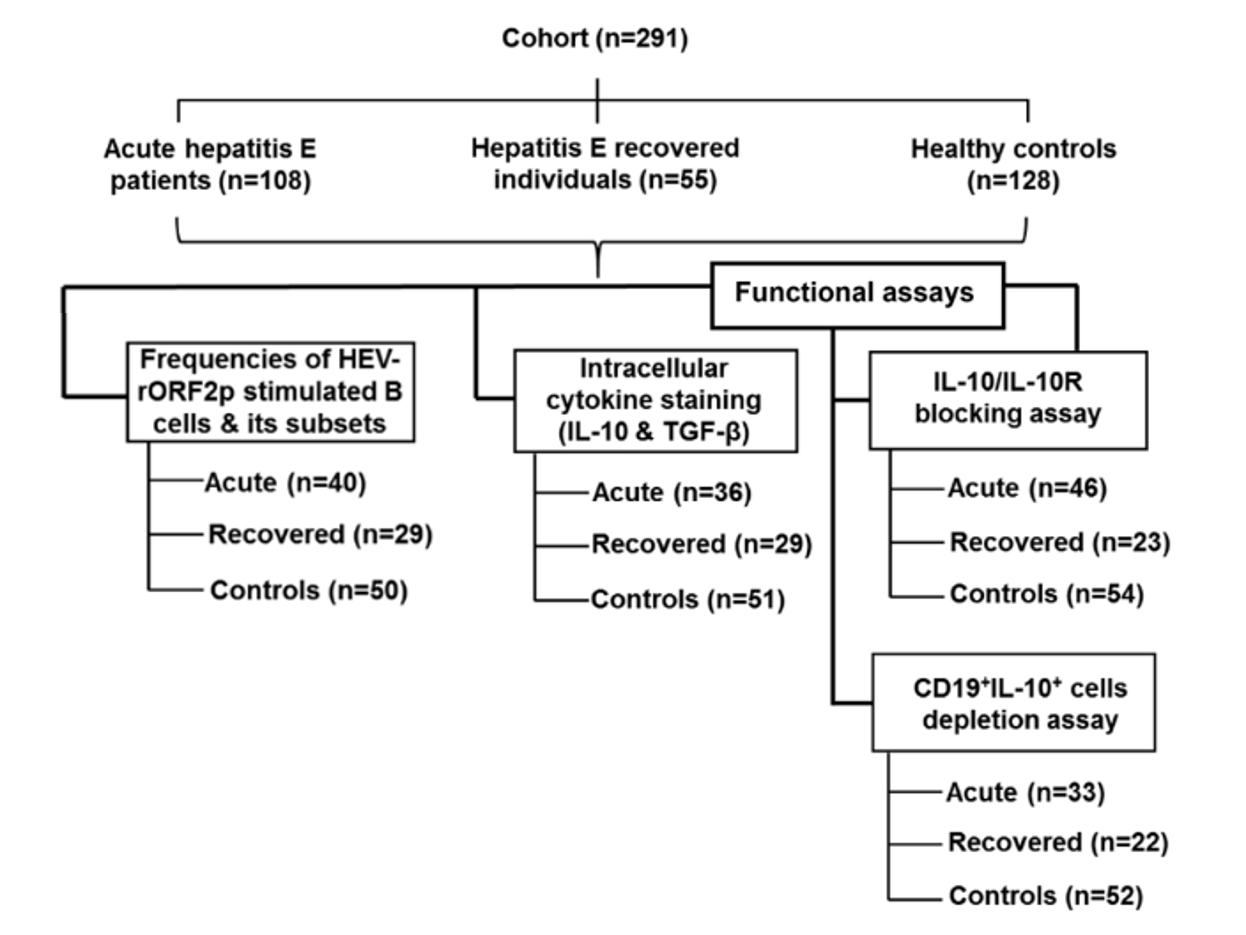
**

**Supplementary figure S1:** Flow chart indicating the number of samples tested in individual assay.

**
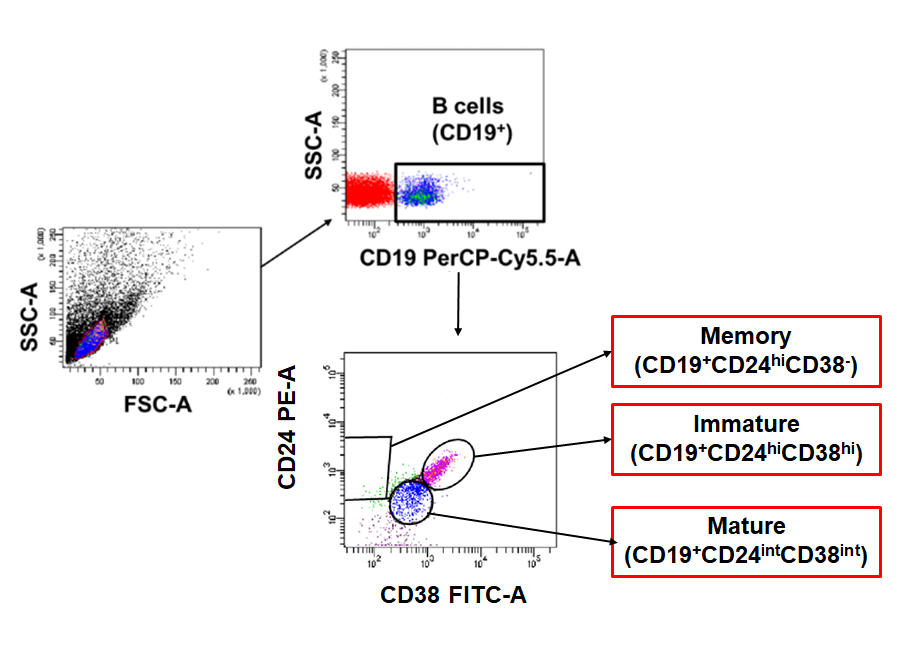
**

**Supplementary figure S2:** Plots showing the strategy used for gating B, immature B, mature B and memory B cells. Lymphocytes were gated from forward vs. side scatter dot plot. B (CD19^+^) were gated from lymphocytes and immature B (CD19^+^CD24^hi^CD38^hi^), mature B (CD19^+^CD24^int^CD38^int^) and memory B (CD19^+^CD24^hi^CD38^-^) cells were gated from B cells.


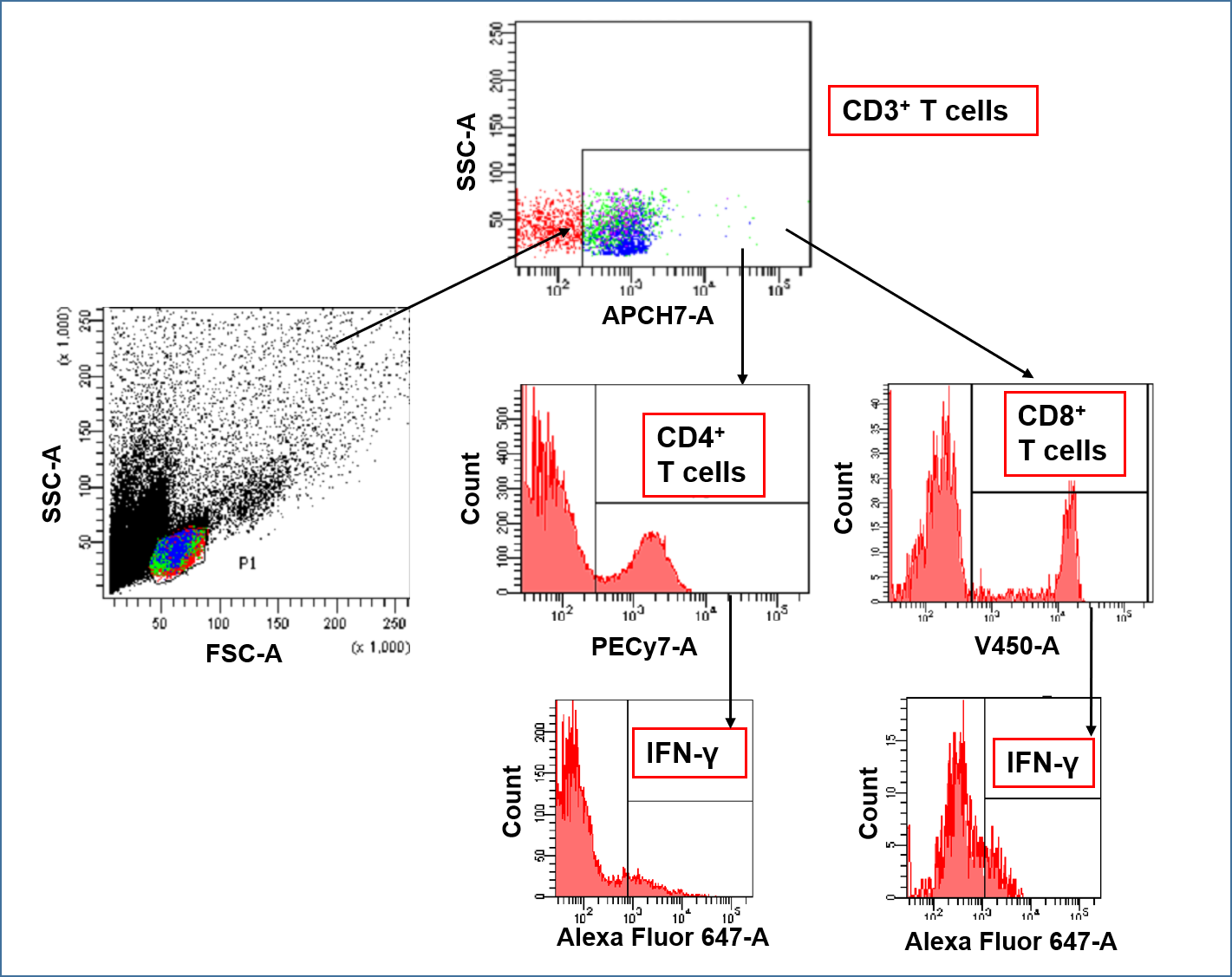
**Supplementary figure S3:** Plots showing the strategy used for gating IFN-γ producing CD4^+^ and CD8^+^ T cells for IL-10/IL-10R blocking assay and CD19^+^IL-10^+^ cells depletion assay. Lymphocytes were gated from forward vs. side scatter dot plot. T cells (CD3^+^) were gated from lymphocytes and IFN-γ producing CD4^+^ (CD3^+^CD4^+^IFN-γ^+^) and CD8 (CD3^+^CD8^+^IFN-γ^+^) T cells were gated from CD3^+^ T cells.
